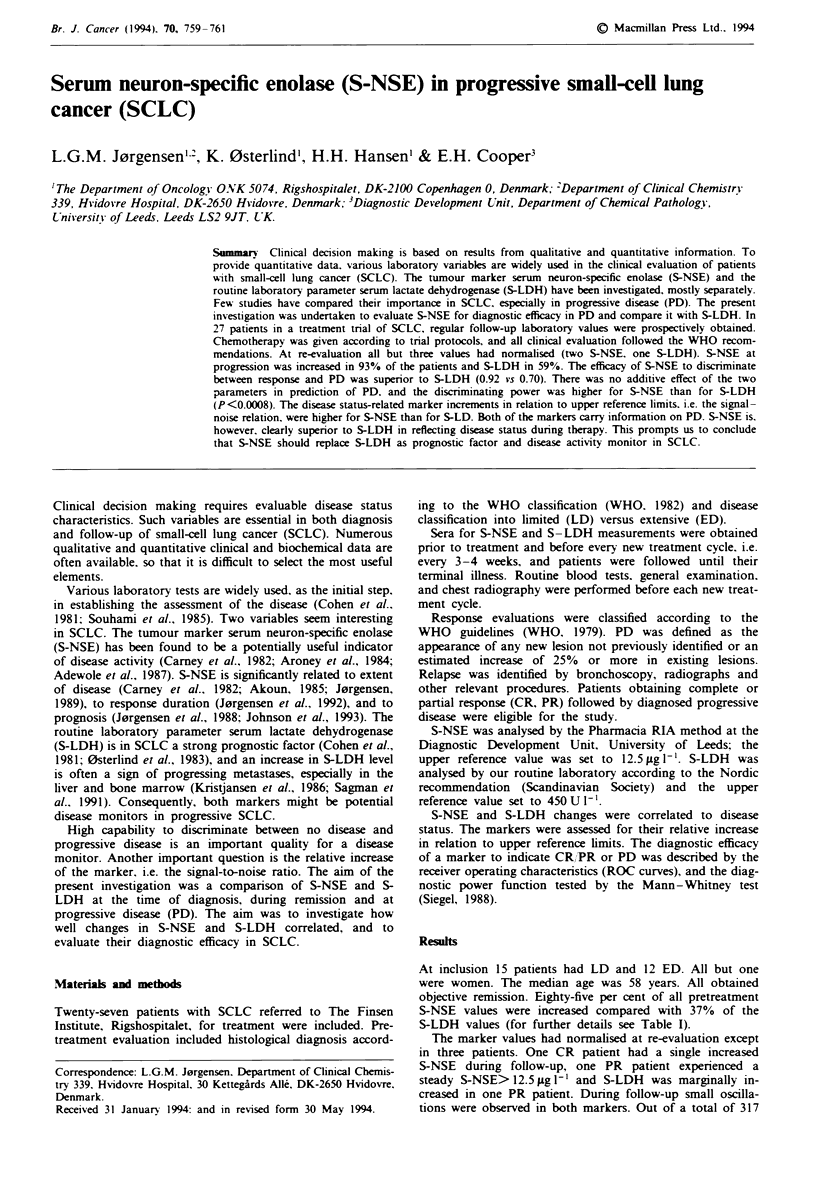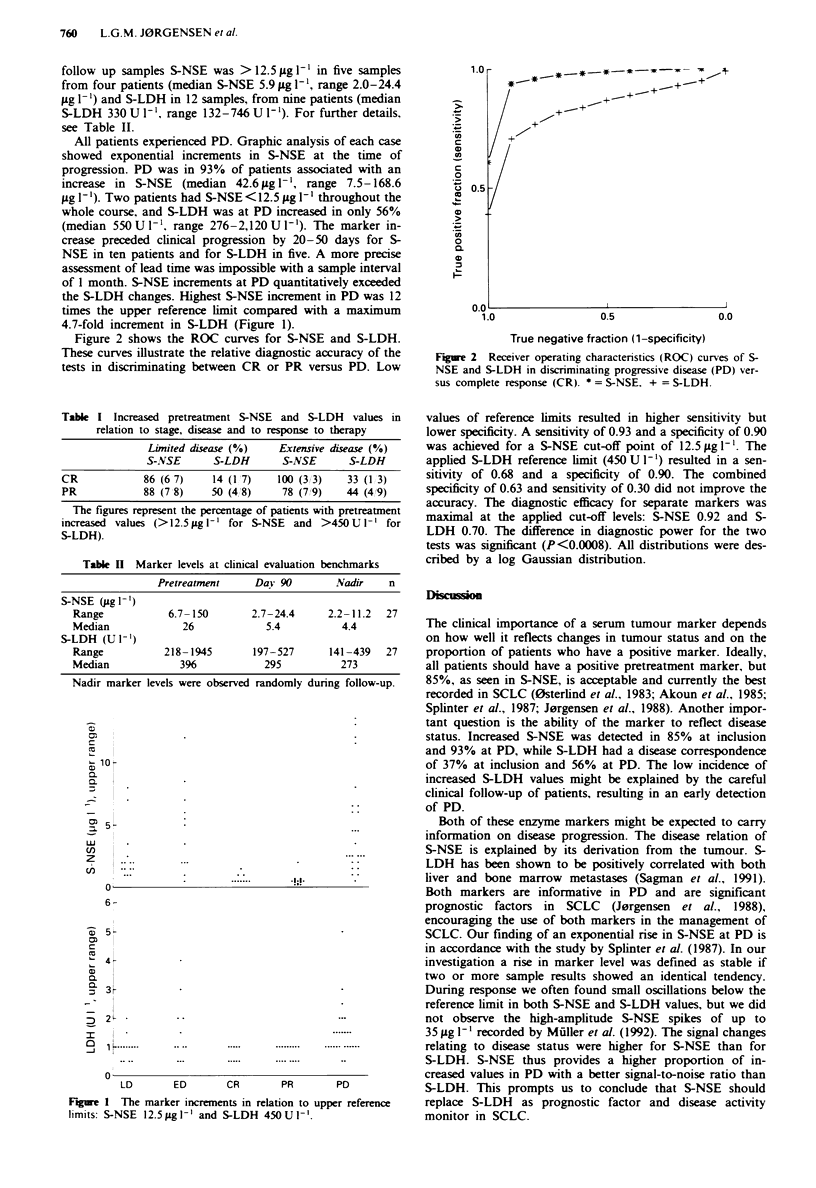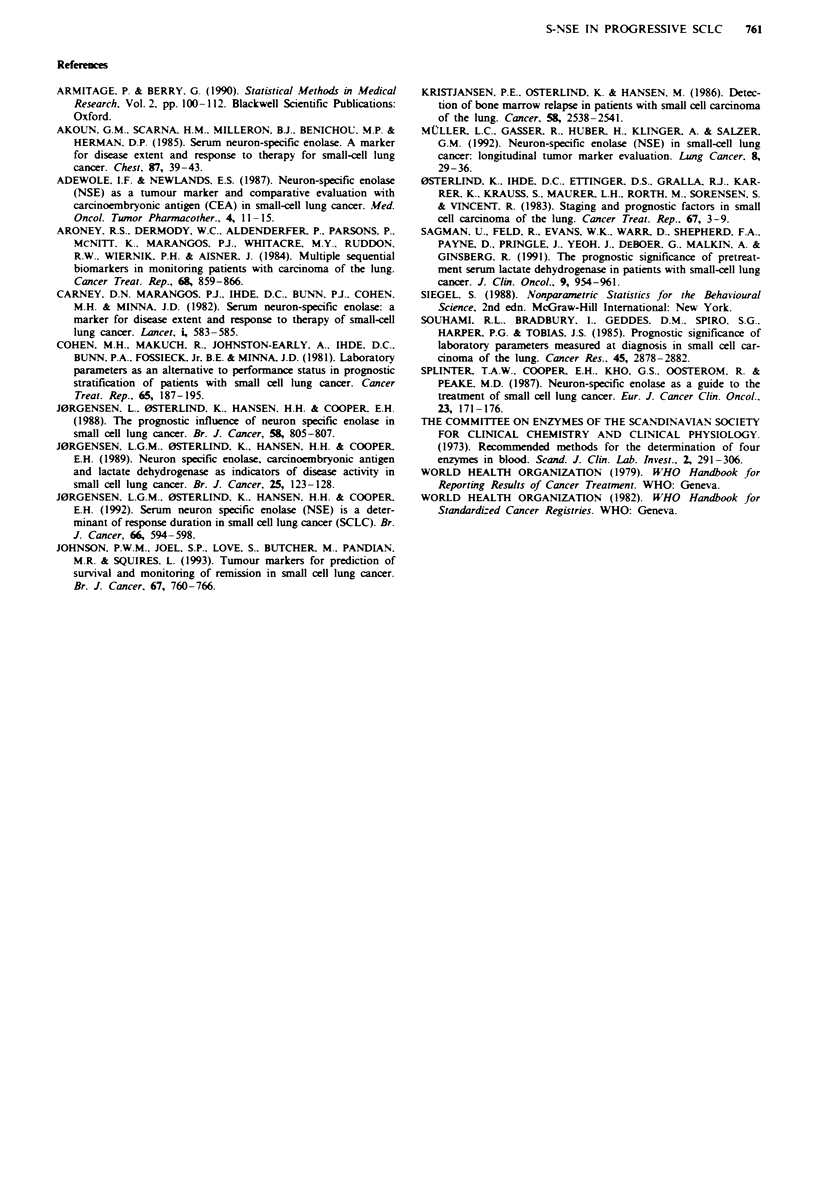# Serum neuron-specific enolase (S-NSE) in progressive small-cell lung cancer (SCLC).

**DOI:** 10.1038/bjc.1994.391

**Published:** 1994-10

**Authors:** L. G. Jørgensen, K. Osterlind, H. H. Hansen, E. H. Cooper

**Affiliations:** Department of Oncology ONK 5074, Rigshospitalet, Copenhagen, Denmark.

## Abstract

Clinical decision making is based on results from qualitative and quantitative information. To provide quantitative data, various laboratory variables are widely used in the clinical evaluation of patients with small-cell lung cancer (SCLC). The tumour marker serum neuron-specific enolase (S-NSE) and the routine laboratory parameter serum lactate dehydrogenase (S-LDH) have been investigated, mostly separately. Few studies have compared their importance in SCLC, especially in progressive disease (PD). The present investigation was undertaken to evaluate S-NSE for diagnostic efficacy in PD and compare it with S-LDH. In 27 patients in a treatment trial of SCLC, regular follow-up laboratory values were prospectively obtained. Chemotherapy was given according to trial protocols, and all clinical evaluation followed the WHO recommendations. At re-evaluation all but three values had normalised (two S-NSE, one S-LDH). S-NSE at progression was increased in 93% of the patients and S-LDH in 59%. The efficacy of S-NSE to discriminate between response and PD was superior to S-LDH (0.92 vs 0.70). There was no additive effect of the two parameters in prediction of PD, and the discriminating power was higher for S-NSE than for S-LDH (P < 0.0008). The disease status-related marker increments in relation to upper reference limits, i.e. the signal-noise relation, were higher for S-NSE than for S-LD. Both of the markers carry information on PD. S-NSE is, however, clearly superior to S-LDH in reflecting disease status during therapy. This prompts us to conclude that S-NSE should replace S-LDH as prognostic factor and disease activity monitor in SCLC.


Br. J. Cancer (1994), 70, 759-761 © Macmillan Press Ltd., 1994

### Serum neuron-specific enolase (S-NSE) in progressive small-cell lung cancer (SCLC)

L.G.M. Jørgensen[1,2], K. Østerlind[1], H.H. Hansen[1] & E.H. Cooper[3]

[1]*The Department of Oncology ONK 5074, Rigshospitalet, DK-2100 Copenhagen 0, Denmark;* [2]*Department of Clinical Chemistry 339, Hvidovre Hospital, DK-2650 Hvidovre, Denmark;* [3]*Diagnostic Development Unit, Department of Chemical Pathology, University of Leeds, Leeds LS2 9JT, UK.*

**Summary** Clinical decision making is based on results from qualitative and quantitative information. To provide quantitative data, various laboratory variables are widely used in the clinical evaluation of patients with small-cell lung cancer (SCLC). The tumour marker serum neuron-specific enolase (S-NSE) and the routine laboratory parameter serum lactate dehydrogenase (S-LDH) have been investigated, mostly separately. Few studies have compared their importance in SCLC, especially in progressive disease (PD). The present investigation was undertaken to evaluate S-NSE for diagnostic efficacy in PD and compare it with S-LDH. In 27 patients in a treatment trial of SCLC, regular follow-up laboratory values were prospectively obtained. Chemotherapy was given according to trial protocols, and all clinical evaluation followed the WHO recommendations. At re-evaluation all but three values had normalised (two S-NSE, one S-LDH). S-NSE at progression was increased in 93% of the patients and S-LDH in 59%. The efficacy of S-NSE to discriminate between response and PD was superior to S-LDH (0.92 *vs* 0.70). There was no additive effect of the two parameters in prediction of PD, and the discriminating power was higher for S-NSE than for S-LDH ($P < 0.0008$). The disease status-related marker increments in relation to upper reference limits, i.e. the signal-noise relation, were higher for S-NSE than for S-LD. Both of the markers carry information on PD. S-NSE is, however, clearly superior to S-LDH in reflecting disease status during therapy. This prompts us to conclude that S-NSE should replace S-LDH as prognostic factor and disease activity monitor in SCLC.

Clinical decision making requires evaluable disease status characteristics. Such variables are essential in both diagnosis and follow-up of small-cell lung cancer (SCLC). Numerous qualitative and quantitative clinical and biochemical data are often available, so that it is difficult to select the most useful elements.

Various laboratory tests are widely used, as the initial step, in establishing the assessment of the disease (Cohen *et al.*, 1981; Souhami *et al.*, 1985). Two variables seem interesting in SCLC. The tumour marker serum neuron-specific enolase (S-NSE) has been found to be a potentially useful indicator of disease activity (Carney *et al.*, 1982; Aroney *et al.*, 1984; Adewole *et al.*, 1987). S-NSE is significantly related to extent of disease (Carney *et al.*, 1982; Akoun, 1985; Jørgensen, 1989), to response duration (Jørgensen *et al.*, 1992), and to prognosis (Jørgensen *et al.*, 1988; Johnson *et al.*, 1993). The routine laboratory parameter serum lactate dehydrogenase (S-LDH) is in SCLC a strong prognostic factor (Cohen *et al.*, 1981; Østerlind *et al.*, 1983), and an increase in S-LDH level is often a sign of progressing metastases, especially in the liver and bone marrow (Kristjansen *et al.*, 1986; Sagman *et al.*, 1991). Consequently, both markers might be potential disease monitors in progressive SCLC.

High capability to discriminate between no disease and progressive disease is an important quality for a disease monitor. Another important question is the relative increase of the marker, i.e. the signal-to-noise ratio. The aim of the present investigation was a comparison of S-NSE and S-LDH at the time of diagnosis, during remission and at progressive disease (PD). The aim was to investigate how well changes in S-NSE and S-LDH correlated, and to evaluate their diagnostic efficacy in SCLC.

### Materials and methods

Twenty-seven patients with SCLC referred to The Finsen Institute, Rigshospitalet, for treatment were included. Pretreatment evaluation included histological diagnosis according to the WHO classification (WHO, 1982) and disease classification into limited (LD) versus extensive (ED).

Sera for S-NSE and S-LDH measurements were obtained prior to treatment and before every new treatment cycle, i.e. every 3-4 weeks, and patients were followed until their terminal illness. Routine blood tests, general examination, and chest radiography were performed before each new treatment cycle.

Response evaluations were classified according to the WHO guidelines (WHO, 1979). PD was defined as the appearance of any new lesion not previously identified or an estimated increase of 25% or more in existing lesions. Relapse was identified by bronchoscopy, radiographs and other relevant procedures. Patients obtaining complete or partial response (CR, PR) followed by diagnosed progressive disease were eligible for the study.

S-NSE was analysed by the Pharmacia RIA method at the Diagnostic Development Unit, University of Leeds; the upper reference value was set to $12.5\,\mu g\,l^{-1}$. S-LDH was analysed by our routine laboratory according to the Nordic recommendation (Scandinavian Society) and the upper reference value set to $450\,U\,l^{-1}$.

S-NSE and S-LDH changes were correlated to disease status. The markers were assessed for their relative increase in relation to upper reference limits. The diagnostic efficacy of a marker to indicate CR/PR or PD was described by the receiver operating characteristics (ROC curves), and the diagnostic power function tested by the Mann-Whitney test (Siegel, 1988).

### Results

At inclusion 15 patients had LD and 12 ED. All but one were women. The median age was 58 years. All obtained objective remission. Eighty-five per cent of all pretreatment S-NSE values were increased compared with 37% of the S-LDH values (for further details see Table I).

The marker values had normalised at re-evaluation except in three patients. One CR patient had a single increased S-NSE during follow-up, one PR patient experienced a steady S-NSE$>12.5\,\mu g\,l^{-1}$ and S-LDH was marginally increased in one PR patient. During follow-up small oscillations were observed in both markers. Out of a total of 317

Correspondence: L.G.M. Jørgensen, Department of Clinical Chemistry 339, Hvidovre Hospital, 30 Kettegårds Allé, DK-2650 Hvidovre, Denmark.
Received 31 January 1994; and in revised form 30 May 1994.

follow up samples S-NSE was >12.5 μg l$^{-1}$ in five samples from four patients (median S-NSE 5.9 μg l$^{-1}$, range 2.0–24.4 μg l$^{-1}$) and S-LDH in 12 samples, from nine patients (median S-LDH 330 U l$^{-1}$, range 132–746 U l$^{-1}$). For further details, see Table II.

All patients experienced PD. Graphic analysis of each case showed exponential increments in S-NSE at the time of progression. PD was in 93% of patients associated with an increase in S-NSE (median 42.6 μg l$^{-1}$, range 7.5–168.6 μg l$^{-1}$). Two patients had S-NSE <12.5 μg l$^{-1}$ throughout the whole course, and S-LDH was at PD increased in only 56% (median 550 U l$^{-1}$, range 276–2,120 U l$^{-1}$). The marker increase preceded clinical progression by 20–50 days for S-NSE in ten patients and for S-LDH in five. A more precise assessment of lead time was impossible with a sample interval of 1 month. S-NSE increments at PD quantitatively exceeded the S-LDH changes. Highest S-NSE increment in PD was 12 times the upper reference limit compared with a maximum 4.7-fold increment in S-LDH (Figure 1).

Figure 2 shows the ROC curves for S-NSE and S-LDH. These curves illustrate the relative diagnostic accuracy of the tests in discriminating between CR or PR versus PD. Low values of reference limits resulted in higher sensitivity but lower specificity. A sensitivity of 0.93 and a specificity of 0.90 was achieved for a S-NSE cut-off point of 12.5 μg l$^{-1}$. The applied S-LDH reference limit (450 U l$^{-1}$) resulted in a sensitivity of 0.68 and a specificity of 0.90. The combined specificity of 0.63 and sensitivity of 0.30 did not improve the accuracy. The diagnostic efficacy for separate markers was maximal at the applied cut-off levels: S-NSE 0.92 and S-LDH 0.70. The difference in diagnostic power for the two tests was significant ($P$<0.0008). All distributions were described by a log Gaussian distribution.

**Figure 2** Receiver operating characteristics (ROC) curves of S-NSE and S-LDH in discriminating progressive disease (PD) versus complete response (CR). * = S-NSE, + = S-LDH.

**Table I** Increased pretreatment S-NSE and S-LDH values in relation to stage, disease and to response to therapy

| | *Limited disease (%)* | | *Extensive disease (%)* | |
|---|---|---|---|---|
| | *S-NSE* | *S-LDH* | *S-NSE* | *S-LDH* |
| CR | 86 (6/7) | 14 (1/7) | 100 (3/3) | 33 (1/3) |
| PR | 88 (7/8) | 50 (4/8) | 78 (7/9) | 44 (4/9) |

The figures represent the percentage of patients with pretreatment increased values (>12.5 μg l$^{-1}$ for S-NSE and >450 U l$^{-1}$ for S-LDH).

**Table II** Marker levels at clinical evaluation benchmarks

| | *Pretreatment* | *Day 90* | *Nadir* | n |
|---|---|---|---|---|
| S-NSE (μg l$^{-1}$) | | | | |
| Range | 6.7–150 | 2.7–24.4 | 2.2–11.2 | 27 |
| Median | 26 | 5.4 | 4.4 | |
| S-LDH (U l$^{-1}$) | | | | |
| Range | 218–1945 | 197–527 | 141–439 | 27 |
| Median | 396 | 295 | 273 | |

Nadir marker levels were observed randomly during follow-up.

**Figure 1** The marker increments in relation to upper reference limits: S-NSE 12.5 μg l$^{-1}$ and S-LDH 450 U l$^{-1}$.

### Discussion

The clinical importance of a serum tumour marker depends on how well it reflects changes in tumour status and on the proportion of patients who have a positive marker. Ideally, all patients should have a positive pretreatment marker, but 85%, as seen in S-NSE, is acceptable and currently the best recorded in SCLC (Østerlind et al., 1983; Akoun et al., 1985; Splinter et al., 1987; Jørgensen et al., 1988). Another important question is the ability of the marker to reflect disease status. Increased S-NSE was detected in 85% at inclusion and 93% at PD, while S-LDH had a disease correspondence of 37% at inclusion and 56% at PD. The low incidence of increased S-LDH values might be explained by the careful clinical follow-up of patients, resulting in an early detection of PD.

Both of these enzyme markers might be expected to carry information on disease progression. The disease relation of S-NSE is explained by its derivation from the tumour. S-LDH has been shown to be positively correlated with both liver and bone marrow metastases (Sagman et al., 1991). Both markers are informative in PD and are significant prognostic factors in SCLC (Jørgensen et al., 1988), encouraging the use of both markers in the management of SCLC. Our finding of an exponential rise in S-NSE at PD is in accordance with the study by Splinter et al. (1987). In our investigation a rise in marker level was defined as stable if two or more sample results showed an identical tendency. During response we often found small oscillations below the reference limit in both S-NSE and S-LDH values, but we did not observe the high-amplitude S-NSE spikes of up to 35 μg l$^{-1}$ recorded by Müller et al. (1992). The signal changes relating to disease status were higher for S-NSE than for S-LDH. S-NSE thus provides a higher proportion of increased values in PD with a better signal-to-noise ratio than S-LDH. This prompts us to conclude that S-NSE should replace S-LDH as prognostic factor and disease activity monitor in SCLC.

**References**

ARMITAGE, P. & BERRY, G. (1990). *Statistical Methods in Medical Research*, Vol. 2, pp. 100–112. Blackwell Scientific Publications: Oxford.

AKOUN, G.M., SCARNA, H.M., MILLERON, B.J., BENICHOU, M.P. & HERMAN, D.P. (1985). Serum neuron-specific enolase. A marker for disease extent and response to therapy for small-cell lung cancer. *Chest*, **87**, 39–43.

ADEWOLE, I.F. & NEWLANDS, E.S. (1987). Neuron-specific enolase (NSE) as a tumour marker and comparative evaluation with carcinoembryonic antigen (CEA) in small-cell lung cancer. *Med. Oncol. Tumor Pharmacother.*, **4**, 11–15.

ARONEY, R.S., DERMODY, W.C., ALDENDERFER, P., PARSONS, P., MCNITT, K., MARANGOS, P.J., WHITACRE, M.Y., RUDDON, R.W., WIERNIK, P.H. & AISNER, J. (1984). Multiple sequential biomarkers in monitoring patients with carcinoma of the lung. *Cancer Treat. Rep.*, **68**, 859–866.

CARNEY, D.N. MARANGOS, P.J., IHDE, D.C., BUNN, P.J., COHEN, M.H. & MINNA, J.D. (1982). Serum neuron-specific enolase: a marker for disease extent and response to therapy of small-cell lung cancer. *Lancet*, **i**, 583–585.

COHEN, M.H., MAKUCH, R., JOHNSTON-EARLY, A., IHDE, D.C., BUNN, P.A., FOSSIECK, Jr, B.E. & MINNA, J.D. (1981). Laboratory parameters as an alternative to performance status in prognostic stratification of patients with small cell lung cancer. *Cancer Treat. Rep.*, **65**, 187–195.

JØRGENSEN, L., ØSTERLIND, K., HANSEN, H.H. & COOPER, E.H. (1988). The prognostic influence of neuron specific enolase in small cell lung cancer. *Br. J. Cancer*, **58**, 805–807.

JØRGENSEN, L.G.M., ØSTERLIND, K., HANSEN, H.H. & COOPER, E.H. (1989). Neuron specific enolase, carcinoembryonic antigen and lactate dehydrogenase as indicators of disease activity in small cell lung cancer. *Br. J. Cancer*, **25**, 123–128.

JØRGENSEN, L.G.M., ØSTERLIND, K., HANSEN, H.H. & COOPER, E.H. (1992). Serum neuron specific enolase (NSE) is a determinant of response duration in small cell lung cancer (SCLC). *Br. J. Cancer*, **66**, 594–598.

JOHNSON, P.W.M., JOEL, S.P., LOVE, S., BUTCHER, M., PANDIAN, M.R. & SQUIRES, L. (1993). Tumour markers for prediction of survival and monitoring of remission in small cell lung cancer. *Br. J. Cancer*, **67**, 760–766.

KRISTJANSEN, P.E., OSTERLIND, K. & HANSEN, M. (1986). Detection of bone marrow relapse in patients with small cell carcinoma of the lung. *Cancer*, **58**, 2538–2541.

MÜLLER, L.C., GASSER, R., HUBER, H., KLINGER, A. & SALZER, G.M. (1992). Neuron-specific enolase (NSE) in small-cell lung cancer: longitudinal tumor marker evaluation. *Lung Cancer*, **8**, 29–36.

ØSTERLIND, K., IHDE, D.C., ETTINGER, D.S., GRALLA, R.J., KARRER, K., KRAUSS, S., MAURER, L.H., RORTH, M., SORENSEN, S. & VINCENT, R. (1983). Staging and prognostic factors in small cell carcinoma of the lung. *Cancer Treat. Rep.*, **67**, 3–9.

SAGMAN, U., FELD, R., EVANS, W.K., WARR, D., SHEPHERD, F.A., PAYNE, D., PRINGLE, J., YEOH, J., DEBOER, G., MALKIN, A. & GINSBERG, R. (1991). The prognostic significance of pretreatment serum lactate dehydrogenase in patients with small-cell lung cancer. *J. Clin. Oncol.*, **9**, 954–961.

SIEGEL, S. (1988). *Nonparametric Statistics for the Behavioural Science*, 2nd edn. McGraw-Hill International: New York.

SOUHAMI, R.L., BRADBURY, I., GEDDES, D.M., SPIRO, S.G., HARPER, P.G. & TOBIAS, J.S. (1985). Prognostic significance of laboratory parameters measured at diagnosis in small cell carcinoma of the lung. *Cancer Res.*, **45**, 2878–2882.

SPLINTER, T.A.W., COOPER, E.H., KHO, G.S., OOSTEROM, R. & PEAKE, M.D. (1987). Neuron-specific enolase as a guide to the treatment of small cell lung cancer. *Eur. J. Cancer Clin. Oncol.*, **23**, 171–176.

THE COMMITTEE ON ENZYMES OF THE SCANDINAVIAN SOCIETY FOR CLINICAL CHEMISTRY AND CLINICAL PHYSIOLOGY. (1973). Recommended methods for the determination of four enzymes in blood. *Scand. J. Clin. Lab. Invest.*, **2**, 291–306.

WORLD HEALTH ORGANIZATION (1979). *WHO Handbook for Reporting Results of Cancer Treatment*. WHO: Geneva.

WORLD HEALTH ORGANIZATION (1982). *WHO Handbook for Standardized Cancer Registries*. WHO: Geneva.